# Characterization and its implication of a novel taste receptor detecting nutrients in the honey bee, *Apis mellifera*

**DOI:** 10.1038/s41598-019-46738-z

**Published:** 2019-08-12

**Authors:** Sooho Lim, Jewon Jung, Ural Yunusbaev, Rustem Ilyasov, Hyung Wook Kwon

**Affiliations:** 1 Department of Life Sciences & Convergence Research Center for Insect Vectors, College of Life Science and Bioengineering, Incheon National University, 119 Academy-ro, Yeonsu-gu, Incheon, 22012 Republic of Korea; 2 Institute of Biochemistry and Genetics, Ufa Federal Research Centre, Russian Academy of Sciences, Ufa, Russia

**Keywords:** Molecular neuroscience, Taste receptors

## Abstract

Umami taste perception indicates the presence of amino acids, which are essential nutrients. Although the physiology of umami perception has been described in mammals, how insects detect amino acids remains unknown except in *Drosophila melanogaster*. We functionally characterized a gustatory receptor responding to L-amino acids in the western honey bee, *Apis mellifera*. Using a calcium-imaging assay and two-voltage clamp recording, we found that one of the honey bee’s gustatory receptors, *AmGr10*, functions as a broadly tuned amino acid receptor responding to glutamate, aspartate, asparagine, arginine, lysine, and glutamine, but not to other sweet or bitter compounds. Furthermore, the sensitivity of *AmGr10* to these L-amino acids was dramatically enhanced by purine ribonucleotides, like inosine-5′-monophosphate (IMP). Contact sensory hairs in the mouthpart of the honey bee responded strongly to glutamate and aspartate, which house gustatory receptor neurons expressing *AmGr10*. Interestingly, *AmGr10* protein is highly conserved among hymenopterans but not other insects, implying unique functions in eusocial insects.

## Introduction

The taste system in animals helps discriminate between harmful, mostly bitter-tasting compounds and nutritious, rich foods that contain sugars or fats (which provide energy) and amino acids (which are building blocks for proteins)^1^. Most animals as diverse as *Drosophila melanogaster* and humans recognize five typical tastes: sweet, bitter, umami (amino acid), salty, and sour (acid). In the past 15 years, gustatory receptors (GRs) for many of the canonical tastes have been identified in a variety of vertebrates and invertebrates^2–5^. In mammals, the attractive sweet and umami tastes are recognized by heterodimeric G protein-coupled receptors of the T1R1, T1R2, and T1R3 complex^6–9^. T1R2 and T1R3 recognize simple sugars, artificial sweeteners, and D-amino acids^6,7^; T1R1 and T1R3 respond to most of the 20 standard amino acids^8,9^. One of the unique characteristics of umami taste is synergism. Purine ribonucleotides including inosine 5′-monophosphate (IMP) and Guanine 5′-monophosphate (GMP) can dramatically enhance the umami taste responses^10^. In insects, a large family of genes encoding G protein-coupled receptors, the gustatory receptor (*Gr*) genes, have been proposed to encode gustatory receptors in the fruit fly^2^, honey bee^11^, mosquito^12^, and silk moth^13^. Subsets of *Gr* genes are expressed in gustatory receptor neurons in the different taste organs, which can discriminate between sweet and bitter tastes^14–19^. Although research in a number of insect species has established detailed mechanisms for detecting various sugars and bitter compounds, taste receptors for standard amino acids are still unknown in insect species, except for IRs of *D*.*melanogaster*^20^.

Perception of amino acids is important taste modality, given that amino acids provide an essential nutrient source for insects, especially egg-laying females^21^. The quality and quantity of amino acids can enhance insect longevity and fecundity^22^. Furthermore, insects prefer sugar solutions enriched with amino acids^23,24^, a behavior that could be mediated by taste receptors. Indeed, the fleshfly and blowfly have labellar sensilla that can respond to amino acids^25,26^, and taste cells in the mosquito and tsetse fly respond to amino acids^27,28^. In *D*. *melanogaster*, the IR76b neurons, which partial overlap with sugar-sensing neurons, in tarsal taste cells can detect amino acids^20^. Also, the labellar taste cells may be specifically sensitive to amino acids^29^, since none of the 18 amino acids tested generated action potentials in the sugar-sensing gustatory receptor neurons^14^.

Like other insects, honey bees also prefer sucrose solutions that include amino acids^30,31^. Foraging honey bees collect pollen to provide the nutrients essential for colony growth and maintenance. A previous study reported that honey bees prefer pollen that is richer in the most essential amino acids^32^, suggesting that pollen amino acid composition affects the foraging behavior of honey bees. Although free amino acids are the second most abundant compounds in nectar, after carbohydrates^33^, it is unknown whether the gustatory receptor neurons of honey bee can recognize amino acids, nor have their GRs for amino acids yet been identified.

Among the insects, the honey bee genome encodes very few gustatory receptors^34^. Based on bioinformatic identification of *Gr* genes in honey bees, *A*. *mellifera* has twelve GRs^34,35^, fewer than in the fruit fly *D*. *melanogaster*^2^, the mosquito *Aedes aegypti*^12^ and the silkworm *Bombyx mori*^36^. Phylogenetic analysis placed *AmGr1* and *AmGr2* in lineages with *D*. *melanogaster* genes encoding sweet-sensing gustatory receptors^37^. Consistent with this, our previous study showed that *AmGr1* responded to sweet substances such as sucrose, glucose, maltose, and trehalose, but not fructose^16^. In addition, phylogenetic analysis showed that *AmGr3* clustered with *DmGr43a* as a fructose receptor in the periphery and a nutrient sensor in the brain^38^. Indeed, it has demonstrated that *AmGr3* responds only to fructose^39,40^ like other *DmGr43a*-like receptors (*BmOr9*^17^ and *HarmGr4*^41^). *AmGr4* and *AmGr5* cluster with *DmGr28a/b* complex^34^, which has been identified in bitter taste neurons in legs^42^ and proboscis taste sensilla^43^. Interestingly, *DmGr28b* control rapid warmth avoidance in *Drosophila*^44^. The high level of homology between *AmGr4/5* and *DmGr28a/b* suggests that they have similar functions. *AmGr11* is included in pseudogenes like *AmGr X*, *Y and Z*^45^. There is not enough information about *AmGr12* because it has been found recently^35^. The remaining bee Grs (*AmGr6-10*) showed no apparent relationships with *DmGrs*, suggesting that these receptors may have unique functions in the honey bee, which might include caste-specific behaviors^46^ and sensing nutrients such as amino acids. A recent field study showed that *AmGr10* influences nursing behavior, which is involved in the division of labor^46^. In addition, *AmGr10* was highly conserved among hymenopteran species, especially eusocial insect species (Fig. S1). This unique role of *AmGr10* in division of labor may depend on the nutrient state of the honeybee society. Therefore, our hypothesis is that *AmGr10* functions as a novel nutrient receptor in honey bees.

The goal of the current study was to identify taste receptors that respond to amino acids in honey bee. We cloned full-length *A*. *mellifera* cDNAs encoding candidate amino acid receptors from honey bee gustatory organs, and found that *AmGr10*, which encodes a conserved gustatory receptor in eusocial insects, was expressed in external and internal organs of the honey bee. Using heterologous expression analysis, we found that *AmGr10* is tuned to a set of L-amino acids, especially L-glutamate and L-aspartate, but not to other compounds such as sweet and bitter substances. Furthermore, inosine-5′-monophosphate (IMP), which is known as a umami-taste enhancer^47^, increased the response of *AmGr10* to these amino acids. Finally, we identified the contact chemo-sensilla responding to L-amino acids in the sensilla chaetica of the galea, which are part of the proboscis, where *AmGr10* was expressed. Thus, we have identified a novel gustatory receptor for L-amino acids in the honey bee, and localized its function in the electrophysiological response of specific mouthparts, providing a powerful platform to decode the gustation of honey bee species.

## Results

### AmGr10 is highly expressed in external and internal organs of the honey bee

**Figure 1:**
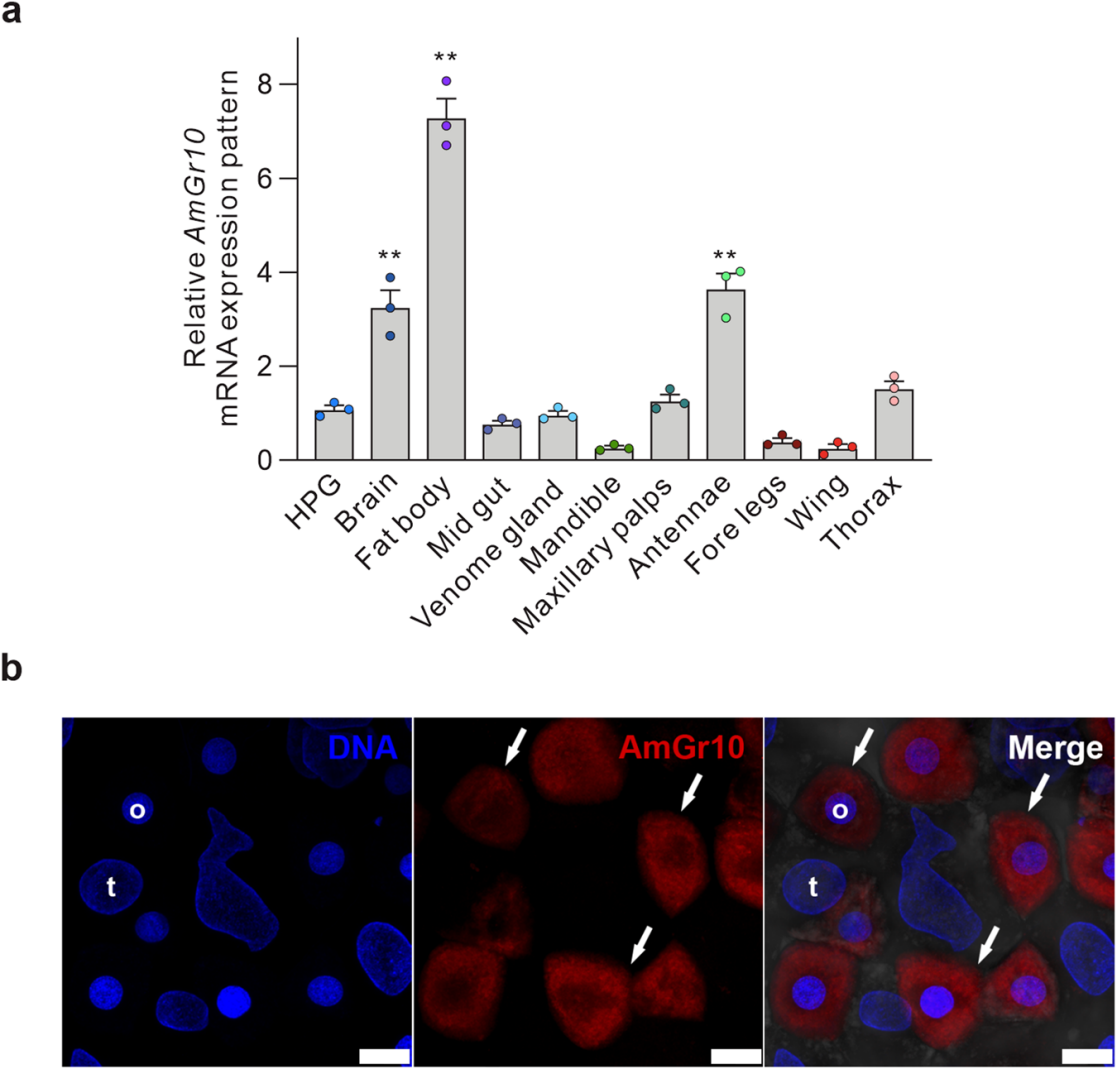
Expression and localization of the gustatory receptor 10 gene of *Apis mellifera*. (**a**) Quantitative real-time PCR analysis of *AmGr10* in organs of worker bees including brain, hypopharyngeal gland, fat body, gut, wing, mandible, maxillary palps, fore legs, thorax, venom gland, antennae (ANOVA with Bonferroni correction. **p < 0.01). Each point represents the mean ± SE. (**b**) Immunostaining with *AmGr10* antibody in fat body tissue of honey bee. *AmGr10* (red, arrows) is expressed only in fat body oenocytes (o), not in trophocytes (t). Scale bars represent 20 µm.

**Figure 2:**
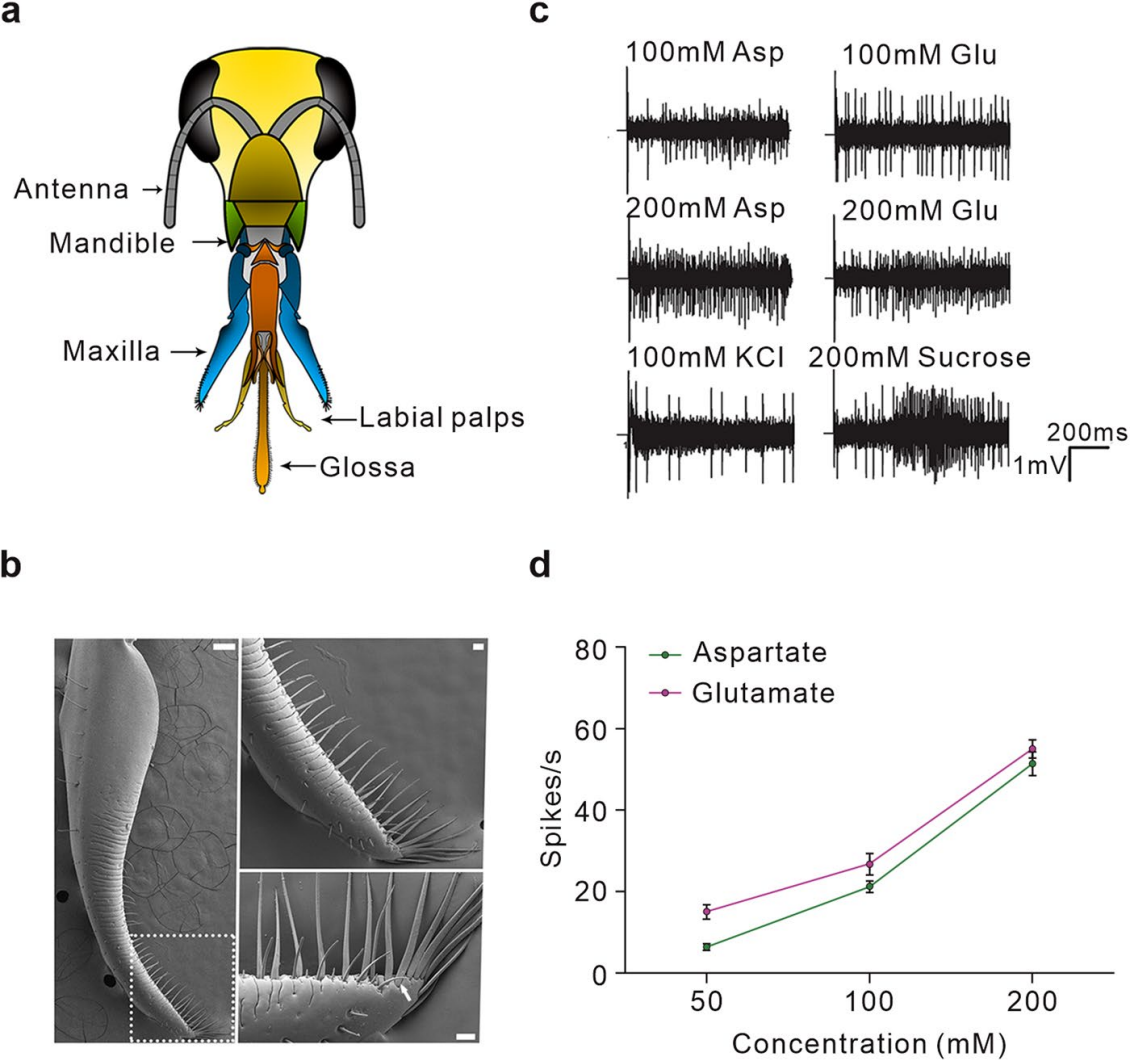
Responses to L-glutamate and L-aspartate in the sensilla chaetica on the galea. (**a**) Mouthparts of honey bee workers consist of mandibles, maxillae, labial palps, and glossa. (**b**) SEM image of a honey bee’s galea of maxilla. The galea of the two maxillae and the labium with two labial palps attached to the glossa. The arrow indicates the first of the ten sensilla chaetica from which tip recordings were made. (**c**) Firing patterns to a series of L-glutamate and L-aspartate concentrations of the sensilla chaeticum in honey bees. For stimulation, 1 mM KCl with sugar concentrations of 100 mM and 1 mM KCl alone were used. (**d**) Chaetic sensilla on the galea respond linearly to the solute concentration of L-glutamate and L-aspartate. Points represent the mean numbers of the responses from an average of 5 hairs per 7 bees. Each point represents the mean ± SE. One-way ANOVA test followed by Bonferroni correction for multiple comparison was employed to test the difference in dose-dependent responses of glutamate and aspartate (**p < 0.01).

**Figure 3:**
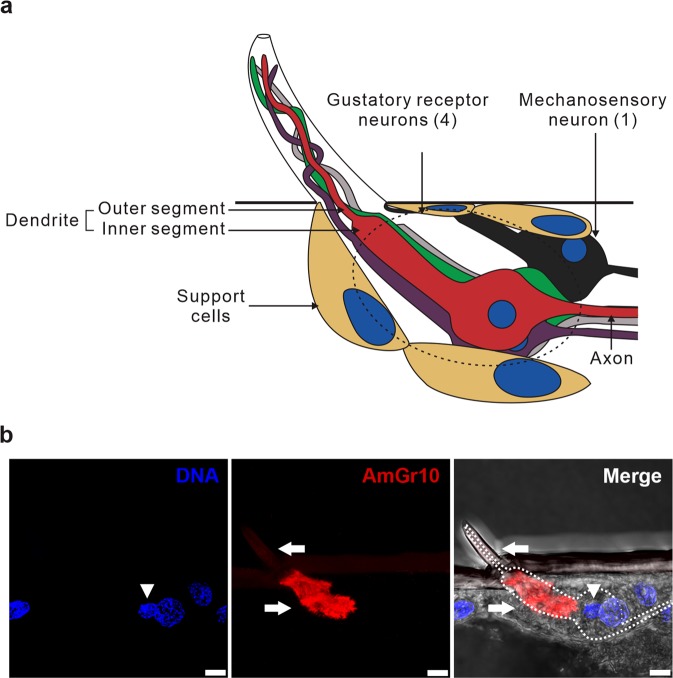
Localization of *AmGr10* in the sensilla chaetica. (**a**) Schematic diagram of the five neurons in sensilla chetica base on previous findings^83^ and this study. There are four gustatory receptor neurons (red, green, purple and grey) and one mechanosensory neuron (black). (**b**) *AmGr10* antibody signal (arrow) was localized to the distal part of galea, where the sensilla chaetica was innervated by *AmGr10*-expressing neurons. The signal was strongly detected in the inner segment of the dendrite and faint in the outer segment. Arrowhead indicates nucleus of *AmGr10*-expressing neurons. Schematic diagram was created with CorelDRAW^®^ Graphics Suite 2019 (Corel Corporation, Canada) by S. Lim.

Expression of *AmGr10* was significantly enriched in gustatory organs, and it was also expressed at high levels in internal taste organs of the gustatory tract including the fat body and hypopharyngeal gland as well as in the brain (Fig. 1a). We confirmed protein expression of *AmGr10* in fat body cells by immunohistochemistry; it was detected in fat body oenocytes but not in trophocytes, which store lipids, protein, and carbohydrates (Fig. 1b). The external sensory organs of the mouthparts are composed of mandibles, maxillae, and labial palps and glossa (Fig. 2a). Each maxilla has a broad, flat plate (the stipe), and the galea, an elongated lobe (Fig. 2b). We found that *AmGr10* localizes to the sensilla chaetica of the galea, which respond to L-amino acids. The proboscis contains various sensilla, which are involved in gustatory processes. Based on previous research showing that a neuron in the sensilla chaetica on the galea may respond to proteins^48^, we investigated if *AmGr10* localizes to the sensilla chaetica, and if these sensilla fire in response to amino acids. Indeed, immunohistochemistry showed *AmGr10*-positive neuron in the pocket of the sensilla chaetica (Fig. 3b; control using pre-immune antiserum in Fig. S4). To assess whether the gustatory sensilla on the galea were sensitive to amino acids, we made tip recordings from the ten most distally located sensilla. The sensilla chaetica on the galea responded to L-glutamate and L-aspartate (Fig. 2b), major components of pollen^49^. Responses to L-aspartate during 1 sec of stimulation ranged from 6 spikes at a concentration of 50 mM, to 22 spikes at 100 mM, and 51 spikes at 200 mM (Fig. 2b,c). Responses to L-glutamate ranged from 15 spikes at 50 mM, to 27 spikes at 100 mM, and 55 spikes at 200 mM (Fig. 2b,c). Two types of gustatory receptor neurons^50^ in this sensilla chaetica had the highest amplitude and response to sucrose stimulation. These results suggest that there is gustatory tuning of amino acids in the sensilla chaetica of the galea.

### Several L-amino acids are ligands for AmGr10

**Figure 4:**
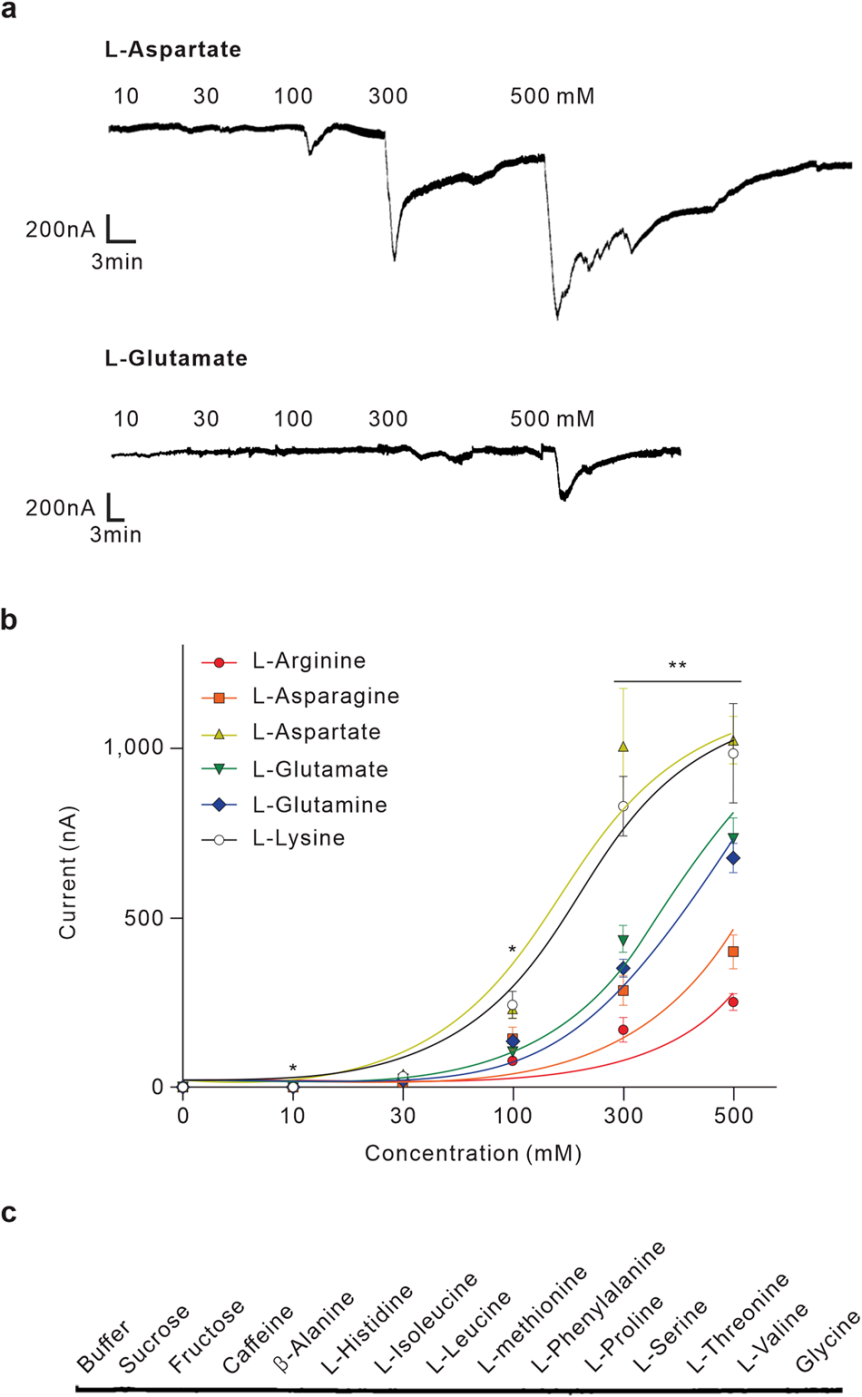
Responses of *Xenopus* oocytes expressing *AmGr10* to stimulation with amino acids. (**a**) Inward current responses of *AmGr10 Xenopus* oocytes stimulated with a range of L-aspartate and L-glutamate concentration at the holding potential of −70 mV. (**b**) Dose-response profile of *AmGr10 Xenopus* oocytes to six amino acids (n = 7). The curve was fitted to the Hill equation. Error bars indicate SE. One-way ANOVA test followed by Bonferroni correction for multiple comparison was employed to test the difference in dose-dependent responses of amino acids (*p < 0.05; **p < 0.01). (**c**) The current traces recorded from *AmGr10*-expressing *Xenopus* oocytes with sequential application of various tested compounds. *AmGr10 Xenopus* oocytes fail to respond to any of the tested sugars, the bitter substances, and some amino acids.

Based on immunohistochemistry and tip recording experiments, *AmGr10* was considered a candidate receptor for L-glutamate and L-aspartate. Using the two-electrode voltage-clamp technique, oocytes injected with *Gr* cRNA were stimulated with L-amino acids. At a holding potential of −70 mV, oocytes injected with *AmGr10* cRNA were responsive to glutamate, aspartate, arginine, asparagine, glutamine, and lysine (Fig. 4a,b), but not to two sugars or a bitter caffeine, nor to other amino acids (Fig. 4c). Based on the dose-response curve of *AmGr10*, the half-maximal effective concentration (EC_50_) values of compounds were 120, 125, 155, 168, 303, and 332 mM for aspartate, lysine, glutamate, glutamine, asparagine, and arginine, respectively. The threshold concentration on *Xenopus* oocytes expressed *AmGr10 in vitro* (~10 mM, Fig. 4c) was slightly lower than that necessary for tip recording L-glutamate and L-aspartate on the honey bee mouthparts *in vivo* (~50 mM, Fig. 2d), suggesting that specific types of L-amino acids were ligands for *AmGr10*.

**Figure 5:**
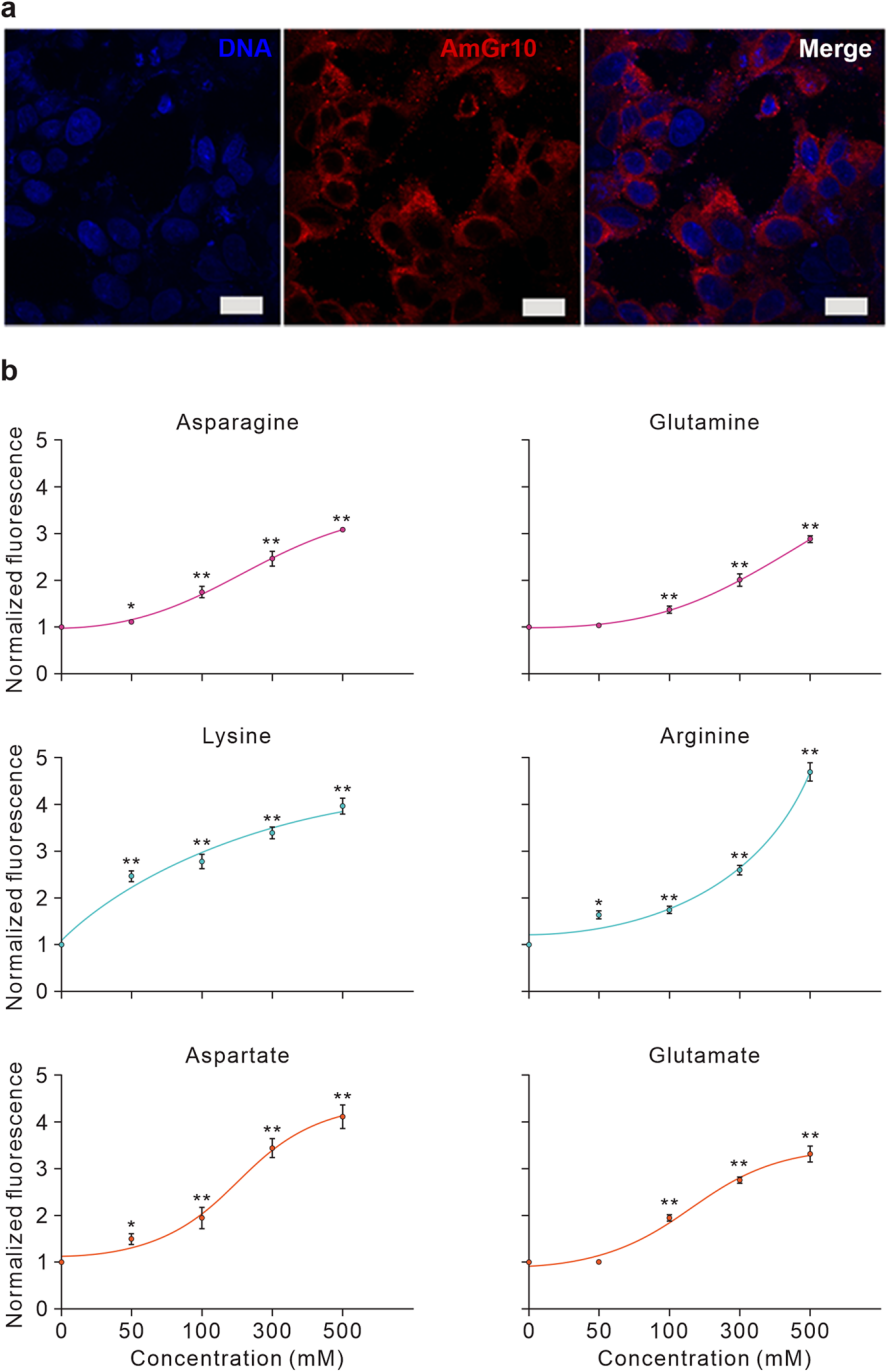
Expression of *AmGr10* protein in cells and Ca^2+^ signaling assay. (**a**) Immunofluorescence of *AmGr10*-expressing HEK 293 cells. The red fluorescence represents *AmGr10*, which indicates apparent staining in the plasma membrane. (**b**) Dose-dependent measurement of intracellular calcium changes using Fluo-4 in HEK 293 cells expressing *AmGr10* and stimulated with 6 amino acids. The Y-axis represents the normalized response, which is shown as the change of fluorescence ratio relative to the ratio of the control (n = 9). Each point represents the mean ± SE. One-way ANOVA test followed by Bonferroni correction for multiple comparison was employed to test the difference in dose-dependent responses of amino acids (*p < 0.05; **p < 0.01).

We also transfected *AmGr10* into HEK 293 cells and performed an intracellular Ca^2+^ concentration assay. Most transfected HEK 293 cells showed *AmGr10* at the cell surface (Fig. 5a; untreated controls shown in Fig. S4). When HEK 293 cells expressing *AmGr10* were stimulated with 100 mM of 17 amino acids, the highest responses were to L-glutamate and L-aspartate, with significant responses also seen to L- arginine, L- asparagine, L-lysine, and L- glutamine (Fig. 5b). This is consistent with two-electrode voltage clamp recording of *Xenopus* oocytes injected with *AmGr10*. Of these amino acids, L-arginine, L-asparagine, L-glutamine elicit a weak umami taste at high concentrations in human sensory tests^51^. The human umami taste receptor, hT1R1/hT1R3, exhibited slight but significant responses to L-Ala, L-serine, L-glutamine, L-asparagine, L-arginine, and L- histidine^51^. In addition, HEK 293 cells expressing *hT1R1/hT1R3* were significantly sensitive to MSG, unlike non-transfected *HEK 293* cells^51,52^. Our experiments using *AmGr10* showed similar result (Fig. S5). These results suggest that *AmGr10* and mammalian umami taste receptors show similar response profiles to L-amino acids.

### Purine ribonucleotides can strongly potentiate the umami taste intensity in AmGr10

**Figure 6:**
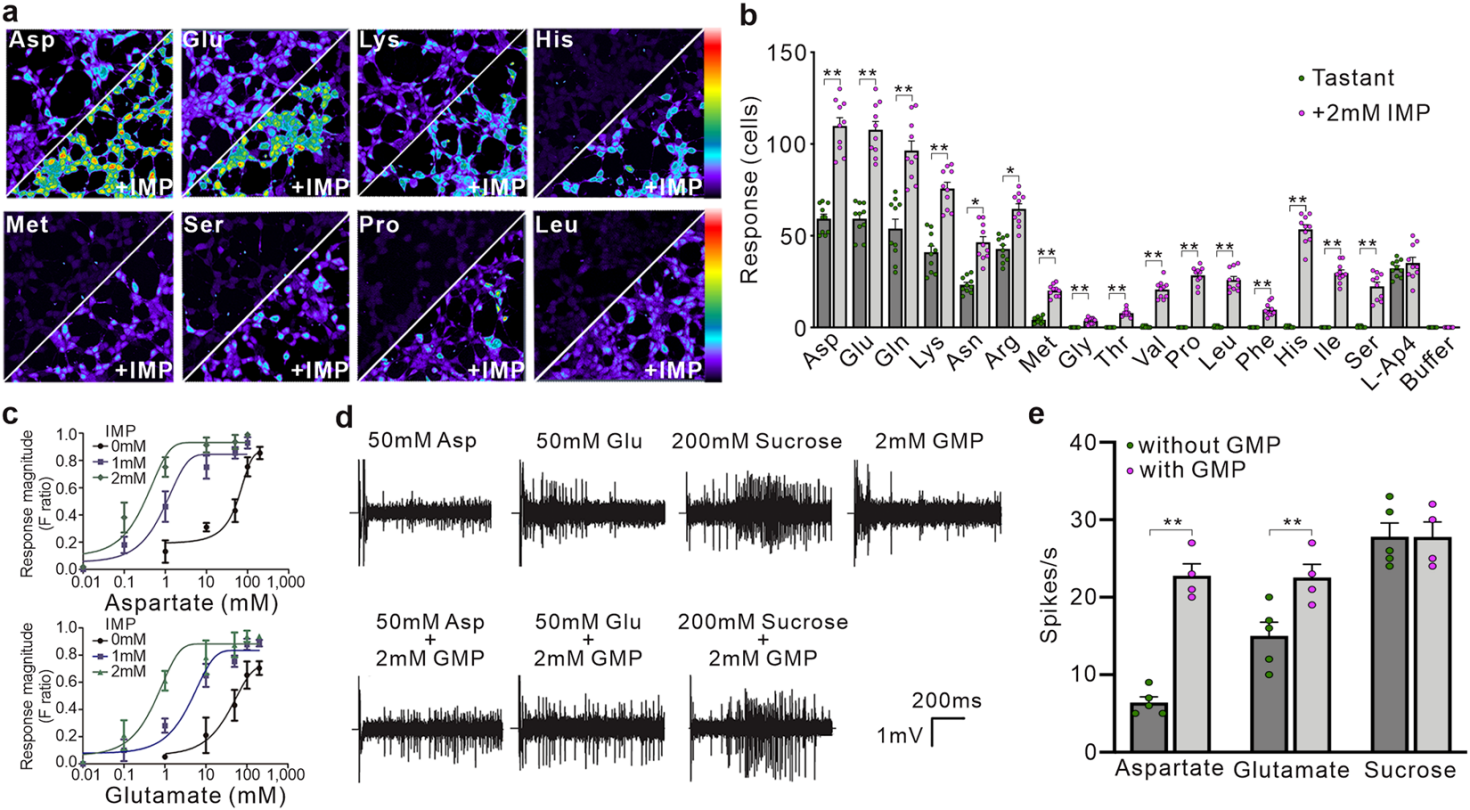
Functional study of *AmGr10* as a receptor for amino acids. (**a**) *In vitro* calcium imaging using HEK cells expressing *AmGr10* showed calcium influx responses to amino acids. HEK 293 cells expressing *AmGr10* were activated by L-amino acids (left) and responses were potentiated by IMP (right). Amino acids were 50 mM and IMP was 2 mM; the color scale indicates the F ratio. (**b**) Quantification of amino acid responses for *AmGr10*. Amino acids were 50 mM and IMP was 2 mM. Each column represents the mean ± SE of at least ten independent determinations. IMP had no effect on *AmGr1* (data not shown). All calcium measurements and quantifications were performed as described in the methods. Significant differences between tastants and combination of IMP and tastants were analyzed using Student’s t test (*p < 0.05; **p < 0.01). (**c**) Dose responses of *AmGr10* to L-aspartate, L-glutamate, and IMP. The presence of 2 mM IMP shifts the responses by at least one order of magnitude to the left (upper EC50 = 0.4 mM, 0.8 mM, and 65 mM, lower EC50 = 0.7 mM, 6 mM, and 70 mM). Each point represents the mean ± SE of ten assays. One-way ANOVA test followed by Bonferroni correction for multiple comparisons was employed to test the difference in dose-dependent responses of glutamate and aspartate with IMP (p < 0.001). (**d**) One-second tip recordings were made from the galeal sensilla. Representative traces of recordings from a chaetic sensillum on the galea of honey bee with the indicated stimuli. (**e**) Honey bees are more sensitive to the stimuli of amino acids with GMP than to amino acids alone. Values represent mean ± SE for n = 5–6 group of bees. Asterisks indicate significant difference by Student’s *t*-test (**p < 0.01).

Previous electrophysiological studies showed that taste responses to L-amino acids can be considerably potentiated by purine nucleotides such as IMP^47^. Also, the mammalian taste receptors T1R1 and T1R3 function as broadly tuned L-amino acid receptors, which sense most amino acids when combined with IMP^8^. Therefore, HEK 293 cells expressing *AmGr10* were stimulated with L-amino acids in the presence or absence of IMP. Relative to controls (Fig. S6), *AmGr10*-expressing cells clearly showed taste responses elicited by six amino acids (Fig. 6a,b and Videos S1, S2) compared to their calcium influx in response to the buffer (Video S5). In addition, low doses of IMP dramatically enhanced the ability of *AmGr10* to sense these L-amino acids (Fig. 6a,b and Videos S3, S4), with effects increasing over 0.1–2 mM (Fig. 6c). However, IMP alone did not activate *AmGr10* (Fig. 5b), even at the highest concentration tested. The effect of IMP on *AmGr10* was saturable (Fig. 6c) and selective; *AmGr1*, a sweet taste sensor^16^, was not activated by L-amino acids in the presence of IMP, nor did IMP enhance the response of *AmGr1* to sweet stimuli (data not shown). In addition, *AmGr10* responses to L-AP4 (a mGluR-agonist) and to other amino acids were greatly enhanced by purine nucleotides (Fig. 6b). Nucleotides such as GMP and AMP are found in floral pollen (Fig. S7), and are thought to enhance umami taste reception^8^. When we stimulated sensilla chaetica of galea with amino acids in the presence or absence of GMP, nerve-firing rates at a given concentration (50 mM) of amino acids were significantly enhanced by 2 mM GMP (Fig. 6d,e). However, GMP had no significant effect on responses to non-amino acid stimulus such as sucrose (Fig. 6d,e). Thus, *AmGr10* is a broadly tuned amino acid receptor that functions as a constituent of the umami response.

## Discussion

### Expression of an umami taste receptor in peripheral and internal organs

*AmGr10*, a GR of the western honey bee *Apis mellifera*, appears to function as an amino acid taste-sensing receptor that is not activated by sweet and bitter compounds. RT-PCR of gustatory receptors in *A*. *mellifera* revealed that expression of seven of nine genes was enriched in gustatory organs such as the labial palps and glossa^34^, but *AmGr10* was not investigated. Recently, Paerhati (2015) reported expression of *AmGr10* in the hypopharyngeal glands, brain, and ovary of nurse bees aged 1 to 14 days, but not in foragers 19 to 29 days^46^. We found that honey bees have *AmGr10*-expressing cells on a variety of structures on the head, body, and legs, as well as being enriched in internal organs such as fat body, brain, and hypopharyngeal gland (Fig. 1a). These internal domains may help monitor amino acid concentration and intestinal absorption or metabolism.

*AmGr10*, in particular, showed a significantly high-expression level in the fat body (Fig. 1a). The fat body is the organ that not only stores fat and glycogen but also synthesizes hemolymph proteins and metabolizes lipids, carbohydrates and amino acids^53^. Therefore, nutrition sensing is essential in the fat body. Indeed *Drosophila* fat body regulates the target of rapamycin (TOR) and the insulin/insulin-like signaling (IIS) pathway secretion directly affecting growth by sensing nutrients, especially amino acids^54,55^. Additionally, *AmGr10* was expressed in the oenocyte, analogue of hepatocyte in mammalian liver^56^, of fat body (Fig. 1b). Nilsen (2011) showed that ILP1 was expressed only in oenocyte, unlike ILP2 was expressed in both oenocyte and trophocyte, and ILP1 was significantly changed in expression pattern according to amino acid supplementation in *Apis mellifera*^57^. Thus, *AmGr10* in fat body may play an important role in the regulation of several physiological mechanisms through the monitoring of amino acid status.

### Comparison of umami responses between mammals and honey bees

In mammals, co-expression of T1R1 and T1R3 is necessary to respond to L-amino acids, whereas *AmGr10* appears not to require the co-expression of other GRs to respond to L-amino acids *in vitro*. However, we cannot rule out the possibility that *AmGr10* is capable of responding to different ligands when combined with other GRs, because *AmGr10*-expressing neurons may co-express other *Grs* among the 12 *AmGr* genes as in the case of the co-expression of *AmGr1* and *AmGr2*^16^.

Heterologously expressed *AmGr10* responds to some polar amino acids. The sensing of amino acids may be through the detection of specific side chains of amino acids; in the honey bee, *AmGr10* detects the carboxylic acids (aspartate and glutamate) and the others containing nitrogen (arginine, lysine, asparagine, and glutamine). Additionally, *AmGr10* responds more strongly to the acidic amino acids than to non-acidic ones, suggesting that recognition of *AmGr10* may depend on its chemical properties. However, the responses of mice T1R1/T1R3 to acidic amino acids are much weaker than to other amino acids^8^, and human T1R1/T1R3 specifically responds to L-glutamate^7^. This suggests that *AmGr10* is more similar to human T1R1/T1R3. Further studies should focus on analyzing the structure of the *AmGr10* using X-ray crystallography to find differences in *AmGr10* ligand specificity, compared to mammals.

### Synergism between L-amino acids and purine ribonucleotides

The sensory properties of umami indicate a synergistic effect between L-amino acids and purine nucleotides such as IMP and GMP^58^. Several studies have revealed numerous candidate umami taste receptors, including T1R1/T1R3^8^, mGluR1^59^, mGluR4^60^, taste-mGluR1^61^, and taste-mGluR4^62,63^. Our results demonstrate that *AmGr10* functions as a broadly tuned amino acid receptor. In addition, *AmGr10*, which showed responses to MSG, L-AP4, and other amino acids, was dramatically potentiated by purine based-5′ ribonucleotides such as GMP and IMP, suggesting strong conservation of basic taste mechanisms between vertebrates and honey bees. We therefore propose that *AmGr10* functions as a constituent of the umami response.

Several research groups have attempted to elucidate a functional domain of umami taste receptors, and found multiple binding sites within the large extracellular Venus flytrap (VTF) domain of mGluRs and T1Rs^64–66^. The VFT domain consists of two lobes and the binding site is located in a hinge region between the two lobes. The L-glutamate binding sites of T1R1/T1R3 localized in the hinge region of the VFT domain in T1R1 and the IMP-binding site is the opening region of the VFT of T1R1^66^. In addition, previous work identified five amino acids that bind to L-glutamate at the hinge region, and these amino acids are conserved between human and mouse^66^. However, It is not yet identified that the VFT domain sequences and five amino acids as critical for L-glutamate binding by *AmGr10*, suggesting that the additional residues critical for L-glutamate and other amino acid recognition in *AmGr10* should still be identified. Future research should focus on determining the binding site of L-amino acids of *AmGr10* using molecular modeling based on the structures of *AmGr10* and site-directed mutagenesis assays.

### Implications of the umami receptor for honey bee social behaviors

Honey bees, which rely on nectars and pollen from flowers, require highly nutritious food to maintain the colony. Thus, detecting amino acids naturally occurring in floral pollen is crucial for the nutrition of honey bees. The concentration of amino acids and crude protein content varies with the pollen’s floral origin over a large range from 4 to 40.8%, with an average of 25%^49,67^. Pollen proteins contain 15 to 19 amino acids, including all essential amino acids, and are particularly abundant in aspartic acid, glutamic acid, glutamine, proline, leucine, lysine, and serine^68,69^. Although the categories of nutritional values that determine pollen quality remain unclear, it is likely that honey bees choose food based on its palatability and nutritional values, using chemosensory organs such as antennae and mouth parts^70,71^. Honey bees can recognize the odor of at least five amino acids (tyrosine, cysteine, tryptophan, asparagine, and proline)^72^. Our finding that *AmGr10* protein was expressed in honey bee mouthparts suggests it may play a critical role in evaluating pollen for amino acids and nucleotides. According to previous studies, as shown in Fig. 3a, of the five neurons located in each galeal chaetic sensilla, one is mechanosensory and the other four respond to tastes. One of the four taste neurons is definitely for sugars, two for electrolytes, and the remaining one is unknown but it may be responsive to amino acids^48^. Using tip recording, we confirmed that some sensilla chaetica in galea were sensitive to umami substances such as L-glutamate and L-aspartate as well as sucrose (sugar substance; Fig. 2b). This may be caused by multiple gustatory receptor neurons in one sensillum^48,50^, but may also be due to the expression of different gustatory receptors in a single neuron cell like mammalian sweet-umami cells^73^. Indeed, *AmGr1*, the sugar taste receptor^16^, is co-localized with *AmGr10* in these type of sensilla (Fig. S8).

Comparing the response threshold of *AmGr10* to amino acids and the concentration of free amino acids in pollen and floral nectar may indicate whether honey bees can recognize amino acids through gustatory receptor neurons housed in the sensilla chaetica. Many studies have reported that the concentrations of individual amino acids in floral nectar rarely exceed 1 mM^74–76^ and are thus lower than the gustatory recognition thresholds in our study. However, the combination of pollen produced in flower handling by foragers may greatly increase the concentration of amino acids in nectar^76^. Indeed, the amino acid content of pollen typically ranged from 14.51–98.93 mM^49^. Interestingly, we found that remarkable synergism between purine ribonucleotides and L-glutamate or L-aspartate occurs in the honey bee gustatory receptors. In addition, in the presence of IMP, HEK 293 cells expressing *AmGr10* responded more strongly to amino acids they did in the absence of IMP. Indeed, we found that GMP is present in floral pollen (Fig. S7), suggesting that honey bees can cope efficiently with a natural range of amino acid concentrations when detecting purine ribonucleotides. Therefore, we conclude that *AmGr10* located on the mouthparts is responsible for the sensitivity of honey bees to a broad spectrum of amino acids through cooperative interaction with purine-ribonucleotides. But like bumble bees, selection for pollen of honey bees might be due to the taste of fatty acids which coated pollen^77^. So further studies are required how the bees can detect the amino acids in fatty acids coat and evaluate the quality of the collected pollen.

### Evolutionary conservation of AmGr10 in eusocial insects

*AmGr10* was highly conserved among hymenopteran species (Fig. S1), but its homologs a distinct GR subfamily that is uncharacteristic compared to other GRs in the sugar or bitter receptor family and is not present in non-eusocial insect species^34,78^, implying that *AmGr10* may have a unique function within hymenopteran insects. A recent study showed that RNA interference-mediated knockdown of *AmGr10* accelerated the transition to foraging^46^, and proposed that *AmGr10* protein is primarily involved in nursing or brood-caring behavior and is thus important in the organization of honey bee societies^46^. Our finding that *AmGr10* responded to six L-amino acids suggests that the *AmGr10* orthologs might be crucial for the perception of amino acids. Although *Drosophila* are known to detect amino acids^29^, the process found was by the IR system (Ir76b), not the Gr system^20^. IR76b has a different tuning range of amino acids from that of *AmGr10*^20^, and *AmGr10* orthologues were not present in the *Drosophila* genome. These unique roles of *AmGr10* for nursing behavior and sensing amino acids may help to explain how the Gr family in eusocial insects regulates honey bee behaviors.

### Concluding remarks

In conclusion, we have provided electrophysiological evidence that honey bee mouthparts respond to amino acids, and identified the amino acid receptor, *AmGr10*, from *Apis mellifera*. Although the insect Grs and mammalian Grs do not resemble each other at the level of amino acid sequences, these gustatory systems have a common mechanism for sensing amino acids in the external environment. Additionally, the amino acid taste receptor was conserved among eusocial hymenopteran insects, suggesting that this *Gr* gene may be a key evolutionary determinant between hymenoptera (especially eusocial) and other orders. Our identification of the amino acid responsiveness of the honey bee GR paves the way for characterizing the other insect GRs and for a better understanding of the contribution of GRs to amino acid perception, and of the modulation of feeding behaviors in insects.

## Materials and Methods

### Insect collection

Foraging honey bee workers were captured near the hive entrance in the morning of every experimental day. To ensure that fully mature workers were harvested, only those that carried pollen or nectar were selected. We estimate their age to range from 21–35 days. The bees were placed in glass vials and cooled on ice until they stopped moving. They were then prepared in the laboratory for molecular and electrophysiological experiments and immunohistochemistry.

### Chemicals

Sucrose (the sugar), the bitter caffeine, the amino acids L-Aspartic acid, L-Glutamic acid, L-Glutamine, L-Lysine, L-Asparagine, L-Arginine, L-Methionine, L-Glycine, L-Threonine, L-Valine, L-Proline, L-Leucine, L-Phenylalanine, L-Histidine, L-Isoleucine, L-Serine, L-Glutamic acid monosodium salt (MSG), the ribonucleotides Inosine 5′-monophosphate (IMP) and Guanosine 5′-monophosphate (GMP) were purchased from Sigma Aldrich (Milwaukee,USA). All the chemicals are of analytical grade (>99.5%).

### RNA isolation, cDNA synthesis and quantitative real-time PCR (qRT-PCR)

Total RNA was isolated from the honey bee antennae using a Qiagen RNeasy Mini Kit (Qiagen, Valencia, CA, USA). Using 1 μg of total RNA, cDNA was synthesized with oligo-dT with Invitrogen Superscript III enzyme (Grand Island, NY, USA). Then, quantitative real-time PCR (qRT-PCR) was carried out with the StepOne Plus (Applied Biosystems, Foster City, CA, USA) using SYBR green qRT-PCR Master Mix (Fermentas, Ontario, Canada) under the following condition: 95 °C for 5 min; 40 cycles of 95 °C for 30 s, 60 °C for 30 s, 72 °C for 30 s. Primers for qRT-PCR are described in Table S1. Quantitative analysis was conducted with StepOne Plus Software V. 2.0 (Applied Biosystems, Foster City, CA, USA). Results were normalized to a validated control gene, Amrps49, using the 2-ΔΔCt method^79^. All biological replicates were conducted in technical triplicate.

### Cloning of the candidate gustatory receptor of *Apis mellifera*

The cDNA synthesis was conducted as described above. For cloning of the candidate gustatory receptor of *Apis mellifera*, PCR amplification was performed using TaKaRa Ex-Taq (Takara Shuzou, Kyoto, Japan) with gene-specific primer sets for the target gene, AmGr10 (XM_006567733.1). Amplification reactions (25 μl) included 0.3 μl TaKaRa Ex-Taq, 2.5 μl 10x Ex-Taq Buffer, 2 μl 2.5 mM dNTP mixture, 2 μl 5 pmol of each primer, 1 μl template cDNA, 17.2 μl sterile distilled water. All amplification reactions were carried out using a 96 Well Thermal Cycler (Applied Biosystems, Foster City, CA, USA) under the following conditions: 95 °C for 3 min, followed by 35 cycles of 95 °C for 30 s, 61 °C for 30 s, 72 °C for 1 min 30 sec, and a final extension at 72 °C for 5 min. PCR amplification products were run on a 1.0% agarose gel and verified by DNA sequencing. Then, the genes that purified by QIAquick PCR Purification Kit (Qiagen, Valencia, CA, USA) were inserted into a pGEM T-Easy Vector (Promega, Madison, WI, USA) using T4 DNA Ligase (Promega, Madison, WI, USA). The recombinant plasmids were transformed into competent *E. coli* (DH5α) cells.

### Heterologous expression of AmGr10 in HEK 293 cells

The expression vector was synthesized by inserting the cDNA of *AmGr10* (*Apis mellifera Gustatory receptor 10*) into the multiple cloning site of the pcDNA3.1 vector using the restriction enzymes EcoRI and NotI (Koscamco, Anyang, Korea). Template pDNA for pcDNA3.1-*AmGr10* and primers were then mixed with kit solutions. The total PCR volume and conditions were followed as described previously^16^. 2.5 µg of the vector with *AmGr10* was transfected into the HEK 293 cells using Lipofectamine 2000 (Invitrogen, CA, USA). The transfection method was according to the manufacturer’s manual. In brief, the Opti-MEM (Invitrogen, CA, USA) was mixed with Lipofectamine at the rate of 6 μL in 125 μL Opti-MEM. It was incubated for 5 min at room temperature. The plasmid DNA (2.5 μg) also was blended with 125 μL Opti-MEM media. These were combined and incubated for 20 min at room temperature. The solution was applied on the HEK cells. After 24 h, the transfected cells were selected using 200 µg/ml zeocin.

### Immunofluorescence analysis

For immunocytochemistry, cells were fixed with 4% paraformaldehyde in PBS for 20 min at room temperature, permeabilized with 0.1% Triton X-100 in PBS (PBST), blocked with 2% normal goat serum and 1% bovine serum albumin (BSA) and labeled with an anti-*AmGr10* antibody (1:500 dilution) at 4 °C overnight. After being washing three times with PBST, cells were incubated with a Cy3 goat anti-rat antibody (1:1000) for 2 h at room temperature. To detect the expression of *AmGr10* in the cells, images were obtained by confocal laser scanning microscopy (LSM700, Carl Zeiss, German) with excitation and emission set to 569 nm and 623 nm, respectively.

### Intracellular calcium assay

For calcium signaling assays, HEK 293 cells expressing *AmGr10* were cultured for more than 3 days, and the Fluo-4 AM dye mix solution (Molecular probes) was loaded into the cell in 96-well microplate. After incubation at 37 °C for 1 hour, the fluorescence signal upon the addition of L-amino acids was measured at 516 nm by excitation at 494 using a spectrofluorophotometer (Perkin Elmer, USA). To normalize the response, the changes in the fluorescence ratio were divided by the maximal fluorescence changes induced by each compound.

### Calcium imaging

Transfected cell lines were subsequently cultured on dishes suitable for confocal microscopy (SPL, Pocheon, Korea). Before each experiment, the culture medium was removed and washed once in Hansk’s balanced salt solution (assay buffer). Transfected cells were loaded with calcium dye Fluo-4 AM (Molecular probes, USA), 2 μM in assay buffer to the dish, after which the cells were incubated in the dark for 90 min at room temperature. After incubation, 1X HBSS buffer was added to the confocal dish, which was then directly placed in the LSM700 inverted confocal microscope for observation (Zeiss, Oberkochen, Germany). Images were captured with a maximum of 100 frames per two-second interval. Test chemicals were dissolved in HBSS at various concentrations. Calcium influx into the transfected cells upon ligand binding was monitored and analyzed with ZEN software (Zeiss, Oberkochen, Germany). For data analysis, response refers to the number of cells responding in a field of about 300 transfected cells^8^. Cells were counted as responders if the F ratio increased above 0.27 after addition of tastants.

### Receptor expression in xenopus oocytes and two-electrode voltage-clamp electrophysiological recordings

Full-length coding sequences of the *AmGr10* cDNA were first cloned into pGEM-T easy vector (Promega, Madison, USA) and then subcloned into the pSDTF vector. *In vitro* transcription of cRNA was performed by using the mMESSAGE mMACHINE SP6 Kit (Ambion, Austin, TX, USA) according to the manufacturer’s protocol. Plasmids were linearized with EcoR1, and capped cRNA was transcribed using SP6 RNA polymerase. The cRNA was purified, resuspended in nuclease-free water at a concentration of 1 μg/μl, and stored at −80 °C in aliquots. Mature oocytes were freed from the follicle cells by treatment with collagenase A for 1 h at room temperature and incubated for 24 hours in modified Barth’s solution^16^. The cRNA was microinjected (27.6 ng) into *Xenopus laevis* oocytes at stage V or VI. The oocytes were then incubated at 17 °C for 3~5 days in Barth’s solution. The two-electrode voltage-clamp technique was employed to observe tastant-induced currents at a holding potential of −70 mV. Signals were amplified with an OC-725C amplifier (Warner Instruments, Hamden, CT), low-pass filtered at 50 Hz and digitized at 1 kHz. Data acquisition and analysis were carried out with Digidata 1322 A (Axon Instruments, Forster City, CA, USA) and software pCLAMP 10 (Molecular Devices, LLC, Sunnyvale, CA).

### Immunohistochemistry

Rat polyclonal antibodies against *AmGr10* were generated by the Abclone company (Seoul, Korea). Based on peptide information of *AmGr10* (GenBank: NP_001229923.1) and the sequence alignment of the peptide, a target epitope region was chosen (NH_2_-SMNTQILIFVCILFLIE-C) to produce polyclonal anti-*AmGr10* antibody. Rats were immunized three times with 0.5 mg of the synthesized *AmGr10* peptide. Serum-specific antibody was purified based on the affinity to immobilized antigen peptides. Prepared honey bee galea samples were directly immersed in 4% PFA and incubated at 4 °C. Samples were washed for 1 h in PBS solution and followed by dehydration through a graded ethanol series of 25, 50, 70, 90, and 100% for 10 min each. In the paraffin embedding process of samples, xylene (Junsei Chemical Co., Japan) was used to efficiently infiltrate paraffin in tissues. Paraffin-embedded preparations of honey bee galea were sectioned at 8-µm thickness by using a microtome (HM340E, Microm, USA). Sections were dried at 40 °C overnight, dewaxed with Citri-Solv (Fisher BioSciences, USA), and rehydrated in an ethanol:PBS series, as described previously^80^. Immunostaining using *AmGr10* antibody was carried out with a blocking solution consisting of 3% normal goat serum in PBS solution. The *AmGr10* antibody was diluted 1:400 in PBS with 0.1% Tween 20 and incubated at 4 °C overnight. After being washed three times with PBST, samples were incubated with a Cy3 goat anti-rat antibody (1:400) for 24 hrs at 4 °C. Samples were washed with PBS solution three times and mounted in mounting medium (Vectashield with DAPI, H-1200, Vector Laboratories, Burlingame, CA, USA). Fluorescent images were captured using a FluoViewFV-3000 confocal microscope (Olympus Corportation, Japan).

### Electrophysiological responses of galeal contact chemosensilla to glutamate and aspartate

Tip recording® experiments were amplified and recorded from the left galea of the proboscis in honey bee mouthparts with IDAC4 with Autospikes software (Syntech, Hilversum, The Netherlands). Bees were immobilized by cooling on ice, restrained in a pipette tip, holding head and appendages in place for electrophysiological measurements. Recordings from gustatory sensilla in galea were made from so-called sensilla chaetica^81^ at the tip of galea devoid of olfactory sensilla. These sensilla can be easily identified by their external morphology^48^. A grounded reference electrode filled with 1 mM KCl was inserted into the compound eye. L-glutamate and L-aspartate were selected for tip recording testing and are common amino acids in plant pollen^82^. For stimulation, 1 mM KCl with sugar concentrations of 100 mM and 1 mM KCl alone were used. Stimuli were applied for approximately 5 s with an inter-stimulus interval of 3 min. Only a few of the taste hairs did not respond to sugar compounds and also did not show mechanoreceptor responses. These sensilla were excluded from the data analysis. The responses of galeal sensilla to all tested solutions were quantified by counting the number of spikes after stimulus onset.

### Extraction of nucleotides in pollen

The procedure for perchloric acid (PCA) extraction was based on previous reports and was conducted as follows: 0.5 g of pollen or beebread was mixed with 10 ml of ice-cold 0.5 M PCA (Sigma-Aldrich). The mixture was incubated at room temperature for 1 hour and then centrifuged (24 °C, 3000 xg, 10 min). The supernatant was transferred to a new microcentrifuge tube, neutralized with 5 M KOH in 1.5 K_2_HPO_4_, and incubated 30 min on ice. To remove potassium perchlorate precipitate, the neutralized samples were centrifuged (24 °C, 3000 xg, 10 min) and filtered with 0.45 µm filter (Millipore).

### HPLC analysis of nucleotides

Analysis of nucleotides was performed based on the methods of previous studies^33^. Preparative high-performance liquid chromatography (HPLC, Ultimate 3000, Thermo Dionex, USA) was used for separation of the constituents from chicken broth. The column was 4.6 mm × 150 mm (C18, Waters, VDS Optilab, Germany) using acetonitrile:methanol:water (1:4.5:4.5, v/v) at a flow rate of 1.5 ml/min and detected at 338 nm.

### Statistical analysis

Relative gene expression and spike counting were analyzed by Student’s *t*-test (SPSS Statistics, Version 25, IBM, NY, USA). Comparisons of voltage clamp responses and spike number were analyzed using a one-way ANOVA test followed by Bonferroni correction with multiple comparisons (SPSS Statistics, Version 25, IBM, NY, USA).

## Supplementary information

Supplementary Information
