# Author Correction: Characterization and its implication of a novel taste receptor detecting nutrients in the honey bee, *Apis mellifera*

**DOI:** 10.1038/s41598-019-53738-6

**Published:** 2019-11-13

**Authors:** Sooho Lim, Jewon Jung, Ural Yunusbaev, Rustem Ilyasov, Hyung Wook Kwon

**Affiliations:** 1 Department of Life Sciences & Convergence Research Center for Insect Vectors, College of Life Science and Bioengineering, Incheon National University, 119 Academy-ro, Yeonsu-gu, Incheon, 22012 Republic of Korea; 2 Institute of Biochemistry and Genetics, Ufa Federal Research Centre, Russian Academy of Sciences, Ufa, Russia

Correction to: *Scientific Reports* 10.1038/s41598-019-46738-z, published online 12 August 2019

This Article contains an error in the Acknowledgements section.

“This study also partly supported by Incheon National University (INU) Research Grant (2016) to H.W.K. and the INU Postdoctoral Fellowships to U.Y. and R.I.”

should read:

“This study also partly supported by Incheon National University (INU) Research Grant (2016) to H.W.K.”

