# Supplementary material for: Characterization and its implication of a novel taste receptor detecting nutrients in the honey bee, Apis mellifera
Source: Sci Rep. 2019 Aug 12;9:11620. doi: 10.1038/s41598-019-46738-z (PMC6690930; doi:10.1038/s41598-019-46738-z)
Supplement: Supplementary file 1 — Supplementary Information [file 41598_2019_46738_MOESM1_ESM.pdf]

Supplementary information

Characterization and its implication of a novel taste receptor detecting nutrients in the honey bee, *Apis mellifera*

Sooho Lim[1,3], Jewon Jung[1,3], Ural Yunusbaev[1,2], Rustem Ilyasov[1,2], and Hyung Wook Kwon[1,*]

[1]Department of Biological Sciences & Convergence Research Center for Insect Vectors, College of Life Science and Bioengineering, Incheon National University, 119 Academy-ro, Yeonsu-gu, Incheon 22012, Republic of Korea

[2]Institute of Biochemistry and Genetics, Ufa Federal Research Centre, Russian Academy of Sciences, Ufa, Russia

[3]These authors contributed equally to this work.

*Corresponding author: Correspondence should be addressed to HW Kwon by e-mail (hwkwon@inu.ac.kr).

Telephone: +82-32-835-8090

Legends for Supplementary information

Figure S1. Phylogenetic relationships of orthologous genes of *AmGr10* in several insect species. Phylogenetic tree was reconstructed based on homologous genes to *AmGr10*.

Figure S2. Control of immunostaining in fat body cells using pre-immune serum of *AmGr10*. Scale bars represent 20 µm. o, oenocyte; t, trophocyte.

Figure S3. Control of immunostaining in sensilla chaetica on the galea using pre-immune serum of AmGr10. Scale bars represent 5 µm.

Figure S4. Control of immunostaining transfected HEK cells expressing *AmGr10* using pre-immune serum of *AmGr10*. Scale bars represent 10 µm.

Figure S5. Differences in response to MSG in *AmGr10*-transfected HEK 293 cells and non-transfected HEK 293 cells (control) by intracellular calcium assay. Unlike control cells, *AmGr10*-trnasfected cells were significantly increased in response to the concentration steps. Each point represents the mean ± SE of triplicate assays. Asterisks indicate significant difference by Student's t-test ($p<0.05$).

Figure S6. Positive and negative controls of calcium imaging analysis using non-transfected, *AmGr1*-, and *AmGr10*-transfected HEK 293 cells to positive chemicals (Calcium ionophore) and to some amino acids which activated AmGr10 as well as buffer solvents. The calcium

ionophore activated both transfected and non-transfected cells (white, positive control). Amino acids did not elicit calcium influx in non-transfected HEK 293 cell lines (n=5).

Figure S7. HPLC profiles of nucleotides in bee bread and mix pollen. Peaks (arrows) represent GMP (Retention Time: 9.13) and AMP (Retention Time: 20.70). IMP was not detected. The concentrations of GMP and AMP in the bee bread sample (0.5% v/v, in distilled water) were 14 μM and 238 μM respectively. In addition, the concentrations of GMP and AMP in the mixed pollen sample (0.5% v/v, in distilled water) were 38 μM and 379 μM, respectively.

Figure S8. Double-immunostaining using *AmGr1* antibody and *AmGr10* antibody. *AmGr10* co-localized with *AmGr1* in sensilla chaetica on the galea. Scale bars represent 5 μm.

Table S1. Primer information of *AmGr10* and *AmRps49*

Video Information (*video files attached in submission system*)

Video S1. A real time calcium imaging for *AmGr10*-expressing cells to 50 μm of L-Glutamate, which clearly showed calcium responses after stimulation.

Video S2. A real time calcium imaging for *AmGr10*-expressing cells to 50 μm of L-Aspartate, which clearly showed calcium responses after stimulation.

Video S3. A real time calcium imaging for *AmGr10*-expressing cells to 50 µm of L-Glutamate with 2 µm of IMP , which clearly showed enhanced response compared to L-Glutamate alone.

Video S4. A real time calcium imaging for *AmGr10*-expressing cells to 50 µm of L-Aspartate with 2 µm of IMP , which clearly showed enhanced response compared to L- Aspartate alone.

Video S5. A real time calcium imaging for *AmGr10*-expressing cells to buffer control solution. No activity was found.

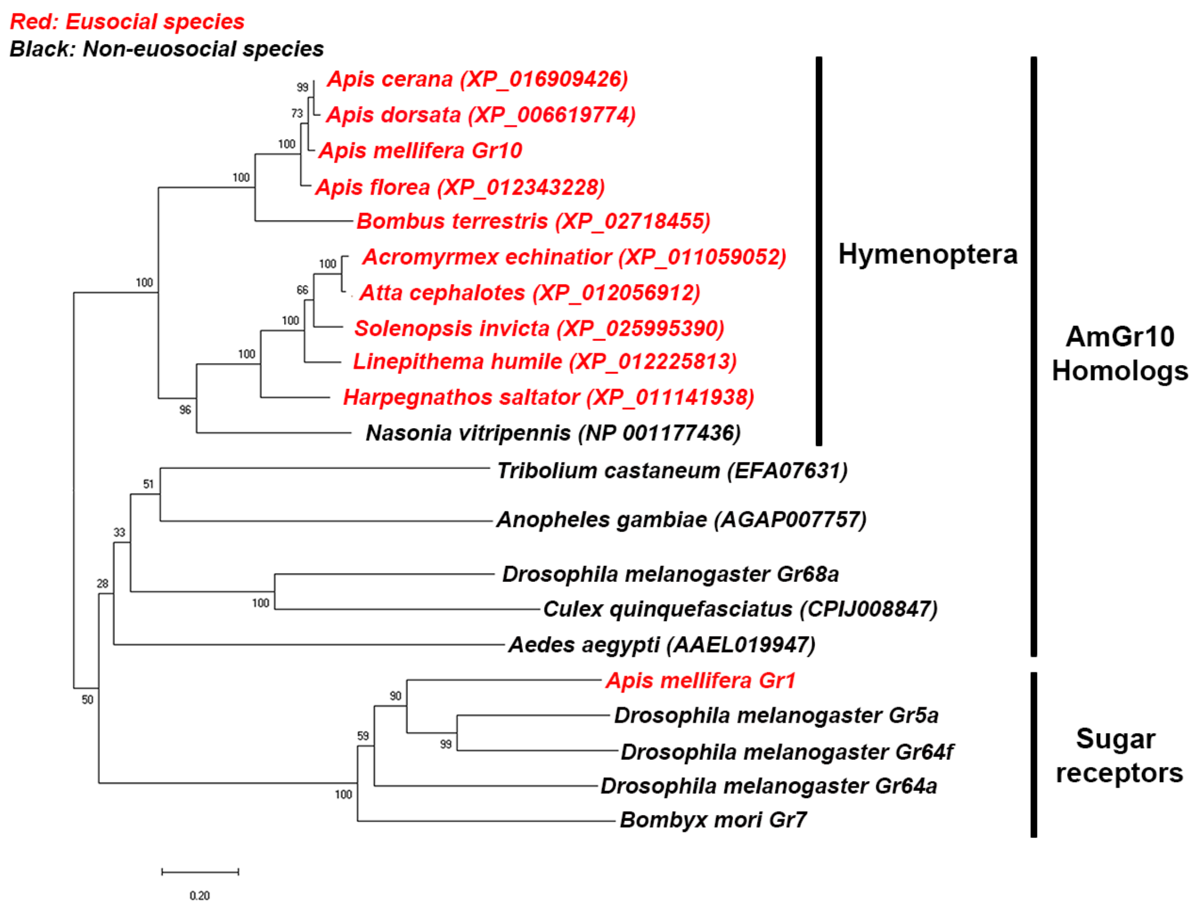
Red: Eusocial species
Black: Non-euosocial species
Apis cerana (XP_016909426)
Apis dorsata (XP_006619774)
Apis mellifera Gr10
Apis florea (XP_012343228)
Bombus terrestris (XP_02718455)
Acromyrmex echinatior (XP_011059052)
Atta cephalotes (XP_012056912)
Solenopsis invicta (XP_025995390)
Linepithema humile (XP_012225813)
Harpegnathos saltator (XP_011141938)
Nasonia vitripennis (NP 001177436)
Tribolium castaneum (EFA07631)
Anopheles gambiae (AGAP007757)
Drosophila melanogaster Gr68a
Culex quinquefasciatus (CPIJ008847)
Aedes aegypti (AAEL019947)
Apis mellifera Gr1
Drosophila melanogaster Gr5a
Drosophila melanogaster Gr64f
Drosophila melanogaster Gr64a
Bombyx mori Gr7
Hymenoptera
AmGr10 Homologs
Sugar receptors
99
73
100
100
100
100
66
100
100
96
51
33
28
100
50
90
59
99
100
0.20

Figure S1

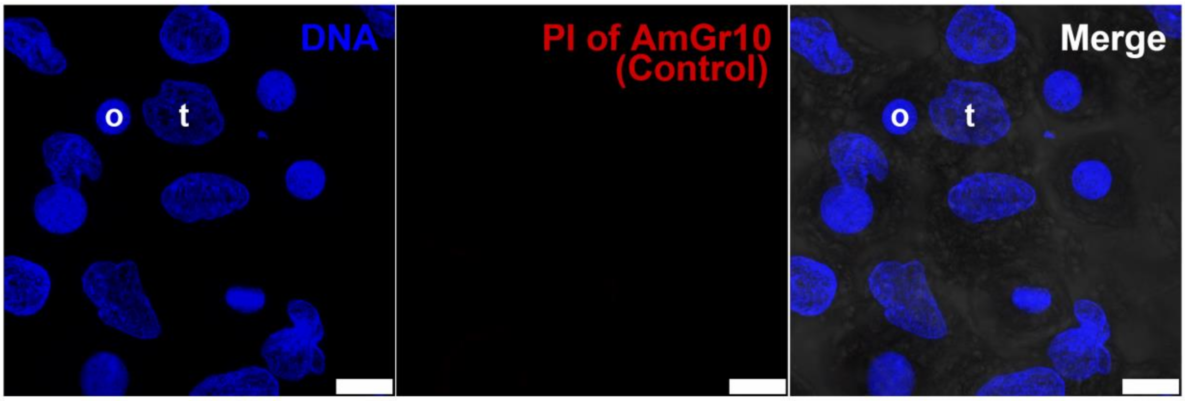

Figure S2

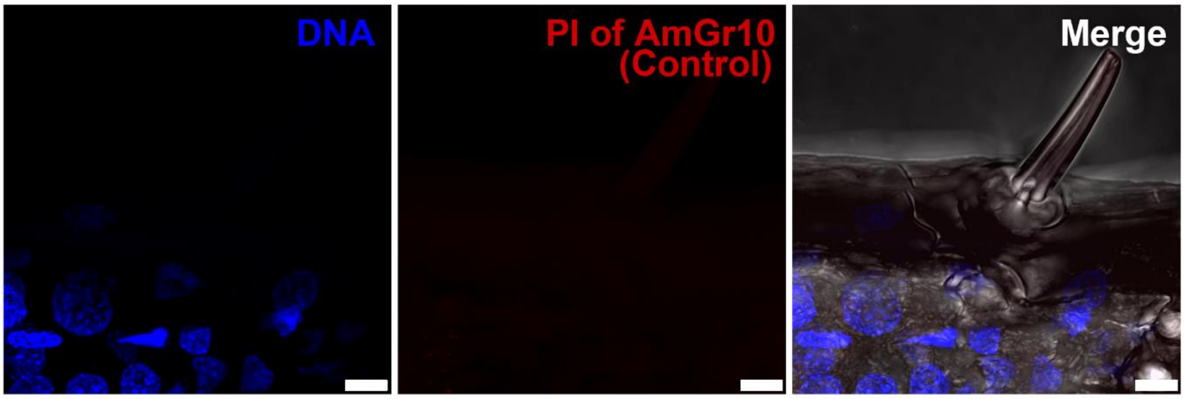

Figure S3

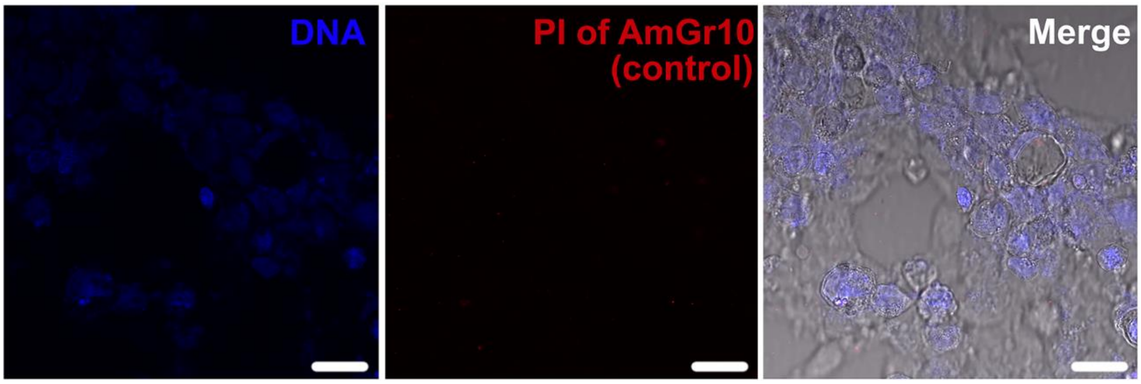

Figure S4

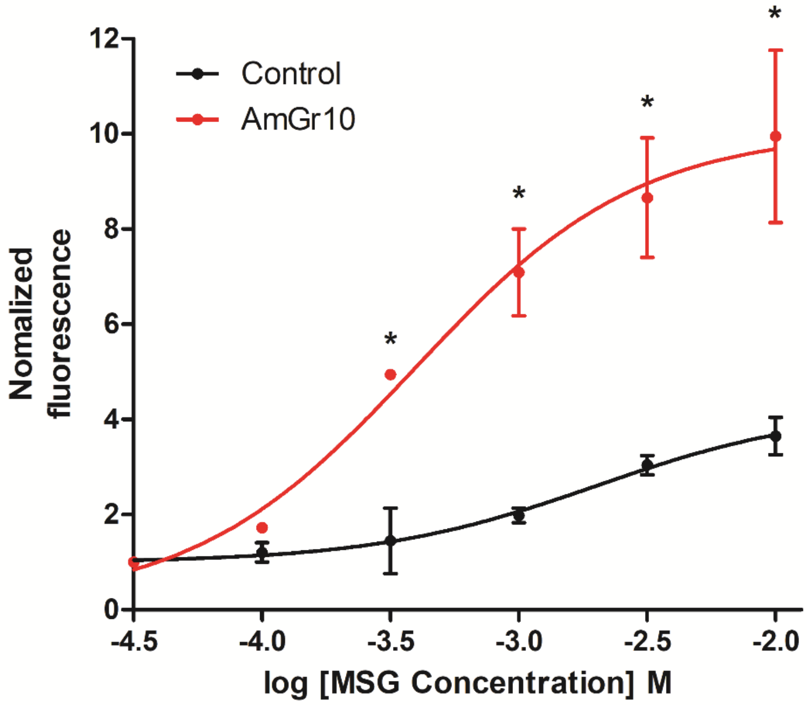

Control
AmGr10
Nomalized fluorescence
log [MSG Concentration] M
12
10
8
6
4
2
0
-4.5
-4.0
-3.5
-3.0
-2.5
-2.0
*
*
*
*
*

Figure S5

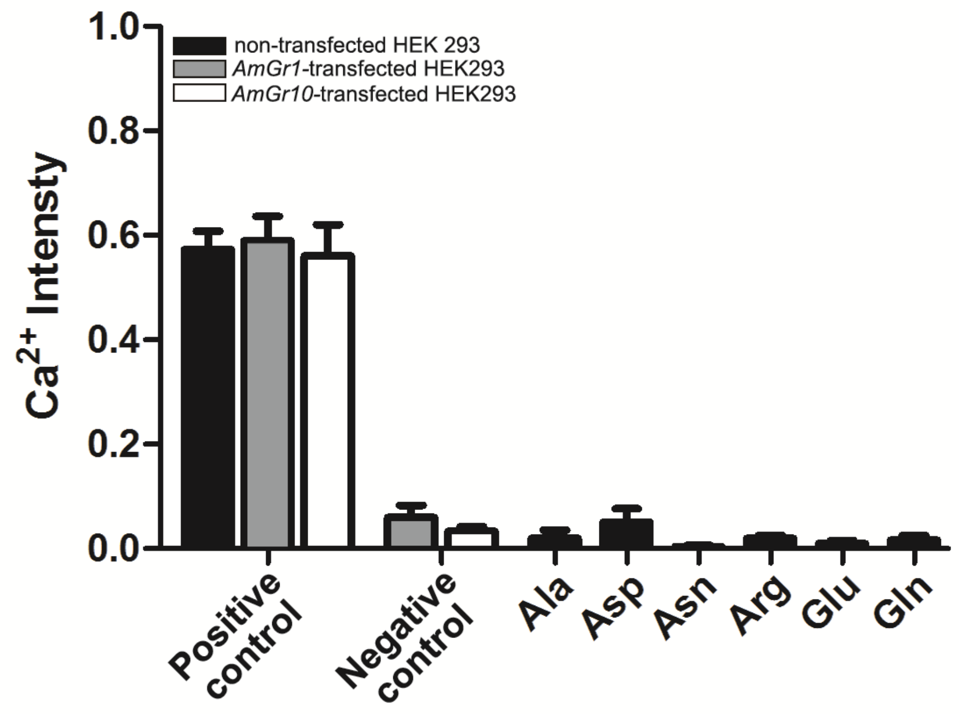
non-transfected HEK 293
AmGr1-transfected HEK293
AmGr10-transfected HEK293
Ca2+ Intensty
1.0
0.8
0.6
0.4
0.2
0.0
Positive control
Negative control
Ala
Asp
Asn
Arg
Glu
Gln

Figure S6

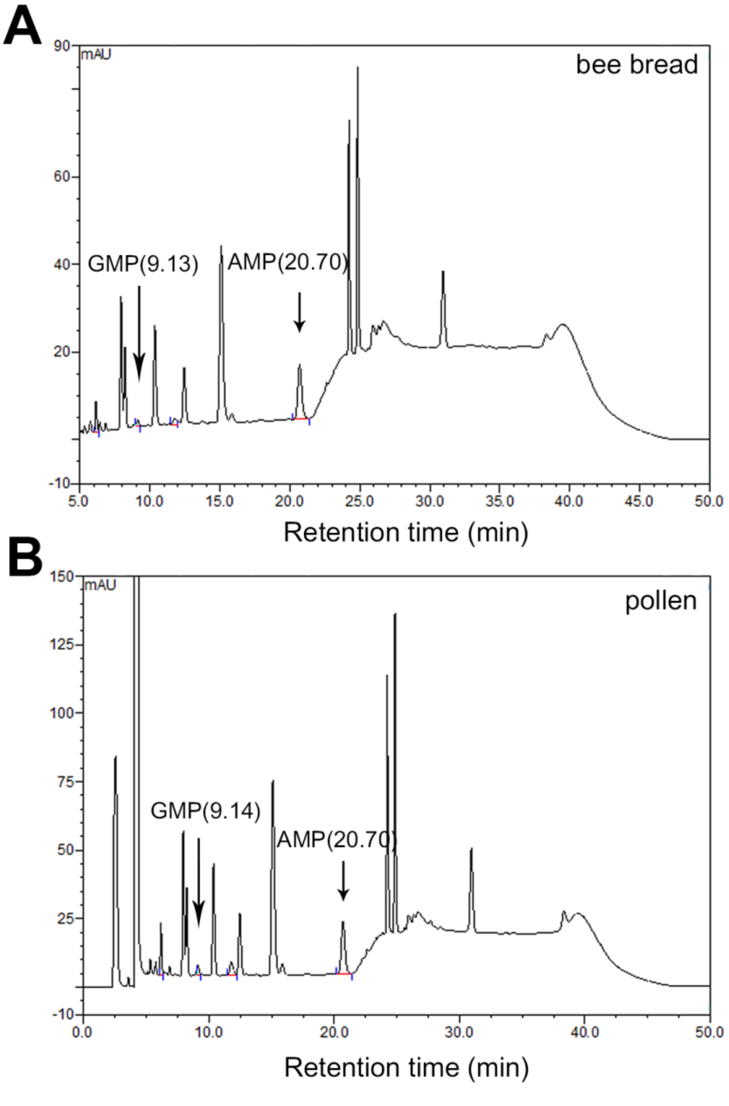
A
bee bread
mAU
GMP(9.13)
AMP(20.70)
Retention time (min)
B
pollen
mAU
GMP(9.14)
AMP(20.70)
Retention time (min)

Figure S7

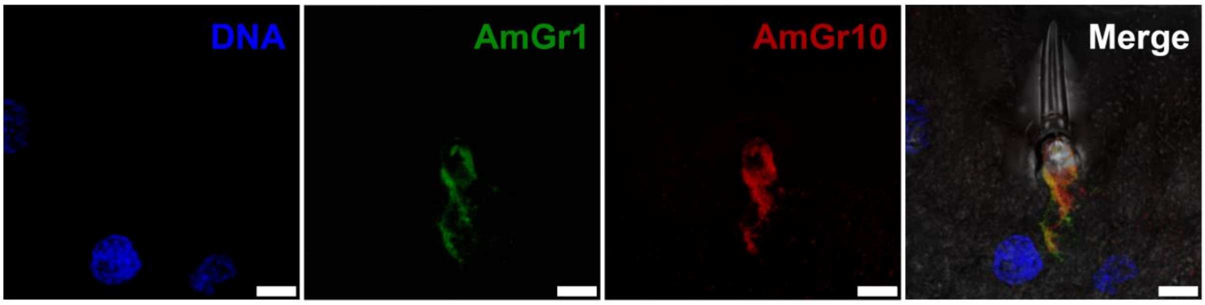

Figure S8

Table S1. Primer information

Gene name	Accession number	Purpose		Primer sequence (5'-3')	Size (bp)
AmGr10	XM_006567733.1	Full-length coding sequence	F	ATGGTGCTCGTTGGAGATACG	1086
			R	TTTTCAATTTCAAATATTTACAACATTTTGGC	
		qRT-PCR	F	AAGCAGCGGCTTACTTTCAA	109
			R	GTGGAAATCTCGTGGAGGAA	
AmRps49	XM_006564316.1	qRT-PCR	F	GGGACAATATTTGATGCCCAAT	100
			R	CTTGACATTATGTACCAAAACTTTTCT	
